# Dosimetry, Toxicity, and Outcomes of Medically Inoperable Endometrial Cancer Treated With Definitive External Beam Radiation Therapy and Brachytherapy

**DOI:** 10.7759/cureus.89982

**Published:** 2025-08-13

**Authors:** Caitlin Reichard, Jeremy Gaskins, Maxwell Kassel, Andres Portocarrero Bonifaz, Scott R Silva

**Affiliations:** 1 Radiation Oncology, University of Louisville School of Medicine, Louisville, USA; 2 Bioinformatics and Biostatistics, University of Louisville, Louisville, USA; 3 Radiation Oncology, Mayo Clinic, Jacksonville, USA

**Keywords:** endometrial cancers, high dose-rate brachytherapy, inoperable uterine cancer, pelvic external beam radiotherapy, radiation gyn, uterine cancer

## Abstract

Purpose: Our goal was to evaluate the dosimetry, toxicity, and outcomes of medically inoperable endometrial cancer patients treated with external beam radiation therapy (EBRT) and brachytherapy.

Methods: A single-institution retrospective chart review was performed to identify patients with medically inoperable endometrial cancer who underwent EBRT and brachytherapy from 2009 to 2024. Patient demographics, tumor characteristics, and radiation dosimetry data were collected. Toxicities were defined using the Common Terminology Criteria for Adverse Events (CTCAE version 5.0). Outcomes evaluated included overall survival (OS) and recurrence-free survival (RFS).

Results: A total of 19 patients were included in the study. Median body mass index (BMI), Charlson comorbidity index, and Karnofsky performance status (KPS) were 50 kg/m^2^, 6.0, and 80, respectively. Median total treatment time with EBRT and brachytherapy was 59 days. Assuming an α/β ratio of 3, the median EQD2 (equivalent dose in 2 Gy fractions) D2cc (minimum dose received by the maximally irradiated 2 cc volume) values to the surrounding organs at risk were 85.9 Gy to the bladder, 64.4 Gy to the small bowel, 69.7 Gy to the sigmoid colon, and 60.1 Gy to the rectum. Less than 20% of patients (n=3) experienced acute or late grade 3 toxicities; 71%, 57%, and 79% of patients experienced no late gastrointestinal, genitourinary, or vaginal toxicities, respectively. In the overall patient cohort, the two-year RFS rate was 67%, and the two-year OS rate was 82%. Cox proportional hazards modeling indicates an association between longer total treatment duration and decreased OS (p=0.046).

Conclusions: Brachytherapy with EBRT can be used to treat medically inoperable endometrial cancer patients with a minimal risk of severe toxicity. OS may be decreased with extended total treatment time.

## Introduction

Endometrial cancer of the uterus is the fourth most common cancer diagnosed in women in the United States and has increased in incidence since 1990, with the incidence increasing most significantly amongst Black women [1]. Risk factors for developing endometrial cancer include obesity, diabetes, polycystic ovarian syndrome (PCOS), nulliparity, and earlier age at menarche. There has been a sustained increase in obese women under 50 being diagnosed with endometrial cancer over the past three decades [1]. Concomitantly, the five-year relative survival rate for uterine cancer decreased from 87% for women diagnosed from 1975-1977 to 81% for women diagnosed from 2013-2019 [2]. 

The standard of care for endometrial cancer is upfront total hysterectomy with bilateral salpingo-oophorectomy with sentinel lymph node dissection, followed by adjuvant treatment depending on the surgical stage. However, a small fraction of patients are poor surgical candidates due to medical comorbidities. These include patients with a high body mass index (BMI), diabetes, advanced age, coronary artery disease, congestive heart failure, pulmonary disease, and hypertension [3]. Ertel et al. reported the incidence of medically inoperable endometrial cancer (MIEC) as approximately 4.6% (35 out of 767 patients with clinical stage I endometrial cancer) [3]. The five-year overall survival (OS) for patients with MIEC is 53.2% [4]. In contrast, the five-year relative survival for all stages of endometrial cancer is approximately 81% [2]. MIEC patients have been treated with definitive hormone therapy and radiation therapy. However, definitive hormone therapy has been associated with worse OS than definitive radiation therapy in MIEC patients, particularly in older patients and those treated at lower-volume centers [5]. Thus, optimizing radiation treatment for this cohort of patients is critical.

A 2015 literature review published by the American Brachytherapy Society (ABS) indicated that while low-dose rate (LDR) brachytherapy was historically used alone or in combination with EBRT for the treatment of MIEC, high-dose rate (HDR) brachytherapy has also shown excellent results when used alone or in combination with EBRT [6]. The ABS supports the use of brachytherapy alone for the treatment of FIGO (International Federation of Gynecology and Obstetrics) stage I, grade 1 or 2, endometrial cancer with minimal myometrial invasion on MRI. The ABS recommends a combination of EBRT and brachytherapy for FIGO stage I endometrial cancer with deep myometrial invasion on MRI, when only CT imaging is available, and for any higher stages.

Implantation quality is also crucial to clinical outcomes. The ABS consensus report indicated that the most common applicator was a vaginal cylinder with single or dual tandems [6]. A 2014 comparison of single, dual, and triple tandem applicators revealed that coverage varies widely depending on the shape of the uterus and its proximity to nearby organs [7]. The Y tandem applicator has been reported to have 62% coverage of the clinical target volume [8]. More recently, Takagawa et al. investigated the use of the Rotte Y applicator vs a three-channel applicator (a combination of the Y applicator and a standard tandem), and the dose to the sigmoid is reduced with a three-channel applicator [9]. However, a drawback of this technique is that it requires more significant cervical dilation, which would not make it suitable for patients with narrow cervices. Furthermore, a triple tandem applicator may not be available in all radiation oncology clinics. Ultimately, an appropriate applicator must be used with consideration of the patient’s gynecological and tumor anatomy.

Given the limited data on brachytherapy applicators for endometrial cancer patients, the objective of this study was to evaluate the dosimetry, toxicity, and outcomes of MIEC patients treated with external beam radiation therapy (EBRT) plus single-tandem or Y-tandem brachytherapy.

## Materials and methods

**Figure 1:**
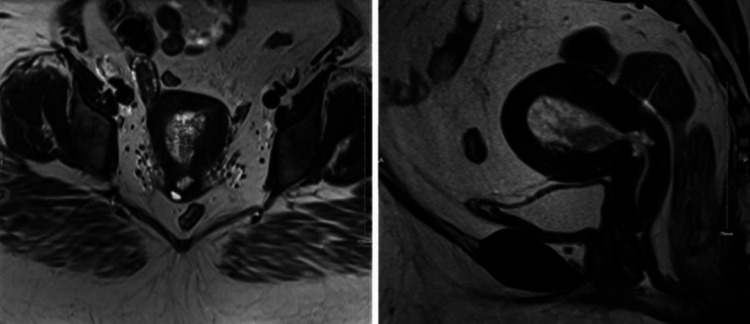
Pelvic MRI of a medically inoperable endometrial cancer patient. T2 pelvic MRI axial (left) and sagittal (right) images of a medically inoperable endometrial cancer in a morbidly obese patient. Due to the concern for cervix involvement noted on the sagittal MRI, this patient was treated with external beam radiation therapy followed by a brachytherapy boost using a Y-tandem applicator.

A retrospective chart review was performed to identify patients with MIEC who underwent EBRT and brachytherapy from 2009 to 2024 at the University of Louisville Brown Cancer Center. Patients treated with brachytherapy alone were excluded from the analysis. Data collected included patient demographics, tumor characteristics, radiation dosimetry, and toxicities. Because endometrial cancer is surgically staged, the T stage could not be determined without an MRI. Before 2020, the Y-tandem applicator used for brachytherapy was not MRI-compatible. Only five patients in our cohort underwent MRI, and these patients could be clinically staged using the FIGO 2009 system (Figure 1) [10]. The National Institute of Health's BMI classification was used, defining normal, overweight, and obese adults as having BMI values of 18.5-24.9 kg/m², 25-29.9 kg/m², and >30 kg/m², respectively. The Karnofsky performance status (KPS) and Charlson comorbidity index were used to quantify our patient population's functional status and comorbidities, respectively.

**Figure 2:**
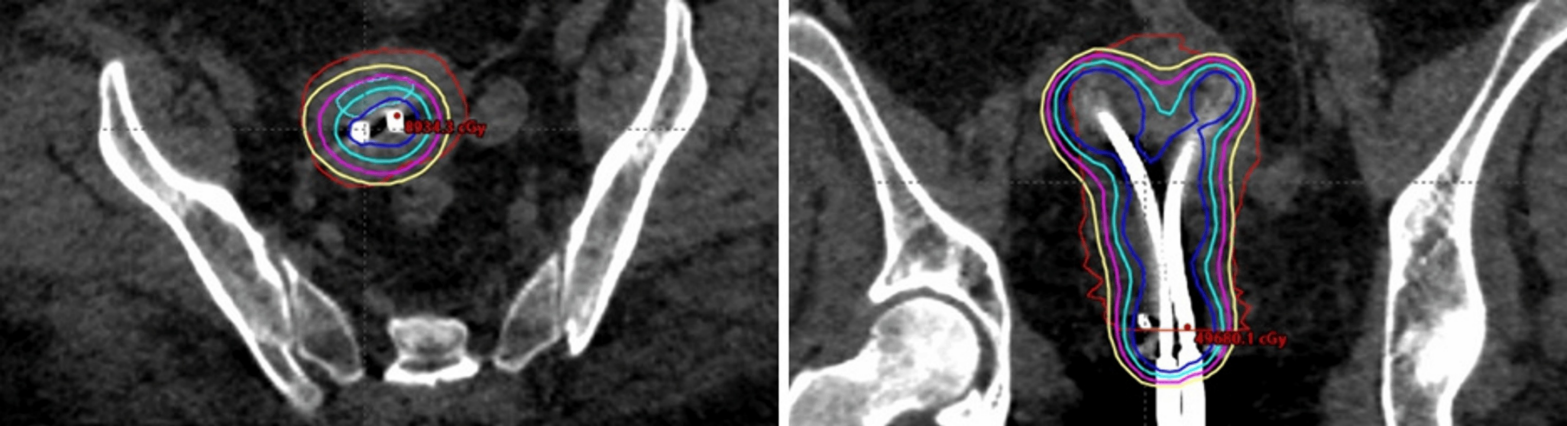
Y-tandem brachytherapy treatment plan. Example of a Y-tandem brachytherapy treatment plan with the Y-tandem shown in the axial (left) and coronal (right) planes of the CT simulation planning scan.  A Y-tandem applicator was implanted in the operating room in a patient with medically inoperable endometrial cancer.  A brachytherapy plan was designed to deliver a dose of 17 Gy in 2 fractions to the uterus.  The prescription isodose line (17 Gy) is depicted in yellow.

CT simulation was performed before EBRT and brachytherapy. The target volume for EBRT included the entire uterus, cervix, proximal vaginal, adnexa, and bilateral pelvic lymph nodes. Regarding the type of EBRT used in our cohort, 12 patients received 3D conformal radiation therapy (3D-CRT), six patients received intensity-modulated radiation therapy (IMRT), and the type of EBRT was unknown in one patient. Brachytherapy applicators were placed under general anesthesia in the operating room, and the target volume for brachytherapy included the entire uterus and cervix. Brachytherapy plans were created in the Varian Eclipse Treatment Planning System using the BrachyVision workspace (Figure 2). Doses to organs at risk (OARs) and HR-CTVs were calculated using the American Association of Physicists in Medicine (AAPM) TG-43 formalism and did not include heterogeneity corrections [11]. Radiation dosimetry data analyzed included absolute EBRT doses and the total equivalent dose in 2 Gy Fractions (EQD2) to the High Risk-Clinical Target Volume (HR-CTV) and the surrounding OARs. Alpha/beta (α/β) ratios of 10 and 3 were used to calculate total EQD2 doses to the HR-CTVs and OARs, respectively. Toxicities were defined using the Common Terminology Criteria for Adverse Events (CTCAE version 5.0) [12]. Outcomes were evaluated via the Kaplan-Meier method and included OS and recurrence-free survival (RFS) after the completion of brachytherapy; univariate Cox regression modeling was used to correlate covariates with these outcomes. All statistical analyses were performed using R statistical software [13]. This study was approved by our institutional review board (IRB 22.0117).

## Results

A total of 19 patients were included in the study (Table 1). The median patient age at the completion of brachytherapy was 64 years (IQR 58-69 years). Median BMI, Charlson Comorbidity Index, and KPS at the initiation of brachytherapy were 50 kg/m^2^ (IQR 35-62 kg/m^2^), 6 (IQR 5-7), and 80 (IQR 70-85), respectively. The number of patients with MIEC increased in the later years of the analysis, with only six patients treated from 2009 to 2017 and the remaining 13 treated from 2018 to 2024. Eighteen patients received EBRT before brachytherapy, and one patient underwent EBRT after brachytherapy. Regarding brachytherapy applicators, 17 patients were treated with a Y-tandem applicator, one was treated with a tandem and ovoids, and one was treated with a tandem and cylinder. All patients were treated with Ir-192 HDR volume-based brachytherapy. Three patients treated prior to 2016 underwent CT simulation for brachytherapy planning, and the prescription isodose line was adjusted to conform to the shape of the uterus without contouring an HR-CTV. The surrounding OARs were contoured for every patient.

**Table 1:** Patient demographics and disease characteristics. BMI = body mass index; HR-CTV = high-risk clinical target volume; KPS = Karnofsky performance scale.

Variable	Median	IQR
Age at treatment completion (years)	64	58-69
BMI (kg/m²)	50	35-62
Charlson comorbidity index	6	5-7
KPS	80	70-85
Tumor size (cm)	2.5	1.7-3.0
HR-CTV volume (cc)	152	103-186

EBRT doses ranged from 33 to 50.4 Gy (in 11 to 28 fractions), with 45 Gy in 25 fractions being the most common EBRT dose regimen. Brachytherapy doses ranged from 14.48 to 34 Gy (in 2 to 4 fractions), with 17 Gy in 2 fractions being the most common brachytherapy dose regimen. The median HR-CTV D90 (minimum dose delivered to 90% of the volume) was 62.8 Gy EQD2 (assuming an α/β ratio of 10) (IQR 57.7-66.2 Gy) (Table 2). Median total treatment time with EBRT and brachytherapy was 59 days (IQR 49-68 days). Radiation dosimetry data (D50 [minimum dose delivered to 50% of the volume], D90, and D98 [minimum dose delivered to 98% of the volume] for the HR-CTV and minimum dose received by the maximally irradiated 0.1 cc volume [D0.1cc], minimum dose received by the maximally irradiated 1 cc volume [D1cc], and minimum dose received by the maximally irradiated 2 cc volume [D2cc] for the OARs) were collected for patients who received EBRT and brachytherapy. Assuming an α/β ratio of 3, the median D2cc EQD2 to the surrounding organs was 85.9 Gy to the bladder, 64.4 Gy to the small bowel, 69.7 Gy to the sigmoid colon, and 60.1 Gy to the rectum.

**Table 2:** Radiation dosimetry data for the entire cohort and for patients who received EBRT plus Y-tandem brachytherapy, including dose to the HR-CTV and doses to the surrounding OARs. HR-CTV = high-risk clinical target volume; EBRT = external beam radiation therapy; OARs = organs at risk; D50 = minimum dose delivered to 50% of the volume; D90 = minimum dose delivered to 90% of the volume; D98 = minimum dose delivered to 98% of the volume; D1cc = minimum dose received by the maximally irradiated 1 cc volume; D0.1cc = minimum dose received by the maximally irradiated 0.1 cc volume; D2cc = minimum dose received by the maximally irradiated 2 cc volume.

Volume parameter	Full cohort			EBRT + Y-tandem		
	Median dose (Gy)	IQR (Gy)	n	Median dose (Gy)	IGR (Gy)	n
HR-CTV D50	85.3	75.8-94.2	16	86.5	81.3-94.2	14
HR-CTV D90	62.8	57.7-66.2	16	63.7	58.8-66.6	14
HR-CTV D98	58.4	54.3-60.0	16	58.8	55.1-60.9	14
D0.1cc - Bladder	110.8	95.6-134.2	19	110.8	92.0-137.3	17
D1cc - Bladder	96.0	78.1-99.3	19	96.0	76.4-99.4	17
D2cc - Bladder	85.9	72.2-90.2	19	85.9	71.6-90.6	17
D0.1cc - Small bowel	77.2	55.3-88.3	19	77.2	55.1-88.3	17
D1cc - Small bowel	68.6	53.2-78.3	19	68.6	52.9-78.5	17
D2cc - Small bowel	64.4	52.4-73.7	19	64.4	52.0-74.3	17
D0.1cc - Sigmoid colon	84.1	80.3-96.1	19	84.1	80.5-98.7	17
D1cc - Sigmoid colon	74.4	68.8-81.0	19	74.9	69.5-81.0	17
D2cc - Sigmoid colon	69.7	64.5-75.0	19	70.1	65.5-75.4	17
D0.1cc - Rectum	68.5	58.8-94.3	19	74.9	61.7-94.8	17
D1cc - Rectum	62.5	54.2-77.1	19	67.3	57.0-78.7	17
D2cc - Rectum	60.1	52.4-71.1	19	64.2	55.6-71.8	17

Table 3 quantifies radiation toxicities following brachytherapy. Acute toxicities were defined as those occurring within six months of completing brachytherapy, while late toxicities were those occurring six months or more after its completion. Most patients experienced no acute toxicities, with 42% reporting no gastrointestinal (GI) or genitourinary (GU) toxicities and 84% reporting no vaginal toxicities. Regarding late complications, 71%, 57%, and 79% of patients experienced no late GI, GU, or vaginal toxicities, respectively. Patients with grade 2 and 3 GI toxicities reported diarrhea >4 stools per day, radiation proctitis, and GI bleeding, requiring transfusions. Grade 2 and 3 GU toxicities included dysuria, difficulty urinating, or incontinence. Patients with grade 2 and 3 vaginal toxicities reported vaginal bleeding; in one case, a patient developed vaginal bleeding about four months after treatment, requiring embolization. Another patient developed vaginal bleeding about seven years after treatment via a rectovaginal fistula. Overall, the majority of patients experienced minimal toxicities.

**Table 3:** Acute and late GI, GU, and vaginal toxicities as defined using the CTCAE (version 5.0). GI = gastrointestinal; GU = genitourinary; CTCAE = Common Terminology Criteria for Adverse Events.

Toxicity	Grade	n	%
Acute GI	No toxicity	8	42
	Grade 1 or higher	11	58
	1	3	16
	2	5	26
	3	3	16
Late GI	No toxicity	10	71
	Grade 1 or higher	4	29
	1	2	14
	2	0	0
	3	2	14
Acute GU	No toxicity	8	42
	Grade 1 or higher	11	58
	1	8	42
	2	3	16
	3	0	0
Late GU	No toxicity	8	57
	Grade 1 or higher	6	43
	1	3	21
	2	2	14
	3	1	7
Acute vaginal	No toxicity	16	84
	Grade 1 or higher	3	16
	1	1	5
	2	0	0
	3	2	11
Late vaginal	No toxicity	11	79
	Grade 1 or higher	3	21
	1	1	7
	2	1	7
	3	1	7

**Figure 3:**
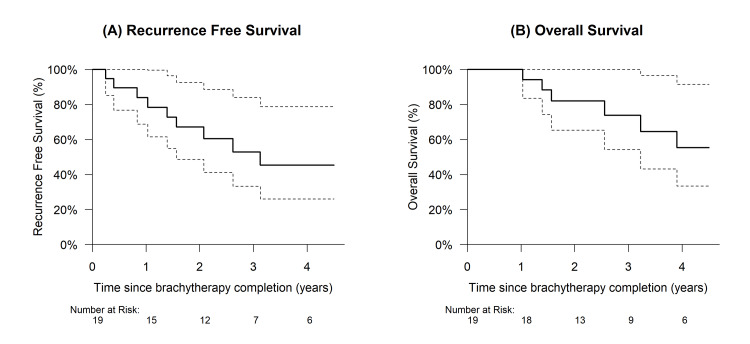
Recurrence-free survival and overall survival. Recurrence-free survival and overall survival of the entire patient cohort. The dashed line indicates the 95% confidence interval.

In the total patient cohort, there were a total of seven observed recurrences (37%), with a two-year RFS rate of 67% (95% CI: 49-93%) (Figure 3A). A total of 10 deaths (53%) were observed, and the two-year OS was 82% (95% CI: 65-100%) (Figure 3B). Cox proportional hazards modeling indicates a statistically significant association between longer total treatment duration and decreased OS (hazard ratio [HR] = 1.17 per 7 days, p = 0.046) (Table 4).

**Table 4:** Univariate hazard ratios for recurrence-free survival and overall survival from Cox proportional hazards regression. HR-CTV = high-risk clinical target volume; HR = hazard ratio.

Recurrence-free survival				
Univariate HRs	HR	95% CI	p-Value	
Total treatment time (days)	1.04	0.90-1.20	0.610	(per 7-day increase)
HR-CTV volume (cc)	1.04	0.95-1.15	0.381	(per 5 cc increase)
Overall survival				
Univariate HRs				
Total treatment time (days)	1.17	1.00-1.37	0.046	(per 7-day increase)
HR-CTV volume (cc)	1.07	0.93-1.22	0.349	(per 5 cc increase)

## Discussion

In this retrospective chart review, we identified patients with MIEC who underwent EBRT and brachytherapy from 2009 to 2024. The dosimetry, toxicity, and outcomes of MIEC patients treated with EBRT plus single-tandem or Y-tandem brachytherapy are then analyzed and discussed in depth. In 2015, the American Brachytherapy Society released a consensus statement detailing the role of EBRT and brachytherapy in the management of MIEC [6]. In 2022, the International Journal of Gynecological Cancer also published a recommendation for curative, definitive radiation therapy for MIEC patients [14]. Our findings add to the evidence to support these consensus statements for MIEC patients. This institutional series analysis highlights the benefits of EBRT + brachytherapy for MIEC patients and delineates the specific workflow details surrounding our practice.

Our results clearly demonstrate that brachytherapy combined with EBRT achieves OS and RFS rates comparable to those reported in the literature. Specifically, our two-year OS rate was 82%, and our two-year RFS rate was 67%, aligning with the two-year OS range of 65.8-84% documented in recent studies [15-22]. Similarly, the 95% confidence interval for the two-year RFS in our study includes previously reported values. For example, a 2021 study reported one- and two-year RFS rates of 89% for brachytherapy alone and 87% and 80%, respectively, for EBRT plus brachytherapy in patients with MIEC [22]. Likewise, our outcomes are consistent with the two-year disease-free survival (DFS) rates of 84% and 79% reported by Rovirosa et al. [23] and Draghini et al. [24], respectively. Furthermore, we report that delayed completion of EBRT and brachytherapy in MIEC significantly decreases OS, similar to studies in cervical cancer and vaginal cancer demonstrating worsened OS with increased treatment duration [25,26].

With respect to toxicity, brachytherapy combined with EBRT remains a key standard of care for MIEC patients, demonstrating favorable safety profiles. An early study conducted between 1965 and 1991 at the Mallinckrodt Institute of Radiology assessed 96 such patients within 30 days of receiving brachytherapy and reported lower life-threatening complication rates compared to surgery [27]. In our study, acute and late GI, GU, and vaginal toxicities were limited, with most patients experiencing either no toxicity or only low-grade events (Grade 0-1) requiring no medical intervention. The most prevalent severe toxicities were late GI events (e.g., proctitis, GI bleeding) and GU symptoms, mirroring findings reported in other studies [4,19,28,29]. Furthermore, a 2017 systematic review by Dutta et al. on MIEC patients treated with radiation alone reported late toxicities ranging from 0% to 21% across 13 studies (encompassing 888 patients) [28], and Van der Steen-Banasik et al. documented a 3.7% incidence of Grade 3 or worse late toxicities for EBRT combined with brachytherapy among 2,694 MIEC patients, compared with 2.8% for brachytherapy alone and 1.2% for EBRT alone [4]. Our findings broadly align with these published estimates, although our small sample size may make our observed proportions appear higher.

Because our cohort of patients had significant medical comorbidities, increasing the risk of serious complications with anesthesia and lying supine for a significant amount of time, the decision was made to treat most patients with brachytherapy to a dose of 17 Gy in 2 fractions, which is a standard treatment option as published by the ABS [6]. With this brachytherapy prescription dose combined with an EBRT dose of 45 Gy in 25 fractions, the EQD2 to the HR-CTV is 70.5 Gy, assuming the entire uterus can be covered. However, due to the proximity of the OARs and limitations of Y-tandem brachytherapy, this target dose usually cannot be achieved without overdosing the surrounding OARS, and a target dose of ~65 Gy EQD2 was deemed clinically acceptable, as a total dose of 65.6 Gy EQD2 is listed as an acceptable dose to the HR-CTV in the ABS consensus statement for the management of MIEC [6]. Regarding dose metrics, the mean HR-CTV D90 and mean D2cc values for the surrounding organs (bladder, small bowel, sigmoid colon, and rectum) exhibit considerable variability in the literature. Reported HR-CTV D90 values range from 45 to 74 Gy [15,19,21,22,30-32], while D2cc values for the bladder, sigmoid, and rectum range from 44 to 90 Gy, 49 to 75 Gy, and 24 to 75 Gy, respectively [15,18,21,22,33]. In our cohort, the median values align well with these published ranges. One possible factor contributing to this variability is the choice of brachytherapy applicator. A 2006 study found that a Y-tandem applicator provided 62% coverage of the clinical target volume [8], highlighting the impact of applicator selection on dose distribution. Similarly, Johnson et al. demonstrated that target coverage varies significantly depending on whether a single-, dual-, or triple-tandem applicator is used, as well as the shape of the uterus and its proximity to adjacent organs [7]. Consistent with these findings, Takagawa et al. reported that a three-tandem applicator provided better dosimetric coverage for large uteri while reducing organ-at-risk toxicity compared to a Y-tandem [9]. These findings emphasize the importance of individualized applicator selection to optimize dose coverage and minimize toxicity. Our department does not have a triple-tandem applicator, which could increase dose conformality to the uterus. We frequently use Syed interstitial brachytherapy in cervical cancer patients to improve dose coverage without increasing toxicity. Because the needles used in interstitial brachytherapy are straight and rigid, for most patients with MIEC that require brachytherapy to the entire uterus, interstitial needles could not safely be placed in the fundus of the uterus without puncturing through the bladder or rectum. The use of steerable brachytherapy needles can solve this problem by being inserted through the vagina and then being steered from the cervix through the uterus to the fundus, and this is currently an active area of investigation in our department [34]. Table 5 provides a summary of key studies on MIEC treated with radiation from 2014 to 2024, reflecting advancements in radiotherapy planning techniques over the past decade.

**Table 5:** Studies from 2014-2024 of patients with medically inoperable endometrial cancer treated with definitive radiation therapy. HDR-ICBT = high-dose rate intracavitary brachytherapy; PRT = pelvic radiation therapy; HR-CTV = high-risk clinical target volume; D90 = minimum dose delivered to 90% of the volume; D98 = minimum dose delivered to 98% of the volume; D2cc = minimum dose to the maximally irradiated 2cc; EQD2 = equivalent dose in 2 Gy fractions; OS = overall survival; FFLP = freedom from local progression; PFS = progression-free survival; DFS = disease-free survival; LRFS = local relapse-free survival; LRRFS = loco-regional relapse-free survival; DMFS = distant metastasis-free survival; CSS = cancer-specific survival; LC = local control; DSS = disease-specific survival.

Reference	Study duration and objective	Total n	Sample description	FIGO stage(s)	Median follow-up time	Dose	Overall survival	Local control	Additional reported outcomes	Toxicity
Rydzinski et al. 2024 [33]	April 2011 and January 2019. Histologically proven EC patients who qualified for definitive HDR-ICBT	78	Median age: 79 years (range: 42-93 years). Median BMI: 39.1 kg/m^2^ (range: 24.2-68 kg/m^2^)	I, II	67 months	HDR-ICBT with 45-52.5 Gy prescribed to HR-CTV in 5-9 fractions given once a week D2cc for OARs: rectum D2cc equivalent dose in 2 Gy (EQD2) ≤ 70 Gy, bladder D2cc EQD2 ≤ 80 Gy, sigmoid colon D2cc EQD2 ≤ 70 Gy.	3-year OS: 69% for whole population. 5-year OS: 55% for whole population	5-year cumulative incidence of local failure: 12.9% (95% Cl: 5.4%-20.5%), 5-year cumulative incidence of distant metastases: 6.4% (95% CI: 0.9%- 11.9%).	5-year cumulative incidence of non-cancer death was 33.1% (95% CI: 22.3%- 43.9%). 5-year risk of cancer death: 9% (95% CI: 3%-16%). 5-year risk of non-cancer death: 36% (95% CI: 25%-47%). No statistically significant impact of BMI ≥ 40 on overall survival (OS) or progression-free survival (PFS). The impact of high-risk features (FIGO II, grade 3 or type 2 cancer) on OS was significant (p = 0.049).	G1 vaginal apex stenosis only.
Carpenter et al. 2023 [17]	December 2015 and August 2020. Patients with MIEC receiving definitive EBRT followed by MRI-based high-dose rate (HDR)-IGBT single institution	32	A total of 75% of patients had FIGO stage I/II disease, 56% endometrioid histology, and 50% grade 3 disease	I, II, III	19.8 months	BT dose was prescribed to HRCTV defined as GTV plus endometrial cavity with a planning goal of a summed EQD2 D90 of ≥85 Gy. HRCTV was prescribed a planning goal of a summed equivalent dose at 2 Gy/fraction (EQD2) D90 ≥85 Gy and D98 ≥65 Gy. An additional intermediate-risk volume (IRCTV) was developed by global 5 mm HRCTV expansion, removing any volume entering into OARs; goal EQD2 D90 ≥70 Gy. EQD2 calculations assumed an α\β of 10 Gy for gross tumor and target volumes and 3 Gy for OARs. OAR constraints: D2cc bladder ≤85 Gy (revised to ≤75 Gy after 2020), D2cc rectum ≤75 Gy (revised to ≤65 Gy in 2016), D2cc sigmoid ≤65 Gy, D2cc bowel ≤65 Gy, and D2cc vagina ≤110 Gy.	12-month OS was 73.6% (95% CI 57.8%-89.3%). 24-month OS was 65.8% (95% CI 48.4%-83.2%)	12-month FFLP was 93.8% (95% CI 85.3%-100%). 24-month FFLP was 88.8% (95% CI 86.6%-91.0%)		23 (72%) patients experienced no RT-related toxicity. 2 of 32 patients (6%) experienced late grade 3+ toxicities (grade 3 refractory vomiting; grade 5 GI bleed secondary to RT-induced proctitis)
Huang et al. 2023 [19]	July 2013 and June 2020. Patients with EC who received definitive radiotherapy	50	Main causes of inoperability were anesthesia contraindications, namely medical comorbidities and obesity.		27 months	EBRT median dose of 45 Gy over 5 weeks. Thereafter, the patients received brachytherapy using tandem and ovoid applicators. Median cumulative D90s in EQD2 to HR-CTV: 72.9 Gy (range, 64.9 to 80.3). Median cumulative D90s in EQD2 GTVp: 166.2 Gy (range, 123.0 to 189.8)	2-year overall survival was 75%	Cumulative incidence of pelvic was 4% (n = 2). Cumulative incidence of distant failure was 16% (n = 8)	8 of the patients died of cancer. 2-year cancer-specific survival rate was 83%.	GI complications of grade 2 or above were noted in 2 patients (4%). Grade 2 GU complication was noted in one.
Rovirosa et al. 2023 [29]	2004-2019 IEC patients receiving EBRT + IGBT in eight European and one Canadian centers	103	Stages: I (44), II (14), III (44). All patients received pelvic ± para-aortic EBRT	I, II, III	28 months (7-170)	Stage I: Median D90-EQD2 (α/β=4.5) to CTV: 73.3 Gy (44.6-132.7). Stage II: Median D90-EQD2 (α/β=4.5) to CTV: 69.9 Gy (44.7-87.9). Stage III: Median D90-EQD2 (α/β=4.5) to CTV: 75.2 Gy (55.1-97)	NA	30 patients relapsed (stages: 10-1, 3-11, 17-III): 24 uterine (stages: 7-1, 3-II, 14-III), 15 nodal (stages: 4-1, 1-11, 10-I). 23 distant (stages: 6-1, 2-II, 15-I).	5-year CSS was 71.2% (stages: 82%-1-ll and 56%-111). 5-year DFS: 55.5%. 5-year LRFS: 59%. 5-year LRRFS: 72%. 5-year DMFS: 67.2%	Late G3-G4 complications (crude): 1.3% small bowel, 2.5% rectum, and 5% bladder.
Rovirosa et al. 2022 [23]	2004 to 2018	62	41 stage-IA and 21 IB HDR-BT was administered in 89%. Spinal anesthesia (38/62) followed by none (16/62) were the most common	IA & IB	32.8 months (SD 33.7)	Median D90 to CTV was 58.9 Gy (8.66-144 Gy).	NA	8 patients presented relapse: 4 uterine, 4 nodal, and 4 distant. 2- and 5-year LRFS was 93.1 and 88.7%. 2- and 5-year LRRFS was 91 and 91%. 2- and 5-year DMFS was 90.2% and 90.2%	2- and 5-year CSS was 93.3% and 80.5%. 2- and 5-year DFS was 84.8% and 80.5%. CSS was better in stage-IA vs. IB (p = 0.043).	Late vaginal G3 and bladder G3-complication rates were 2.1%, respectively.
Shen et al. 2023 [21]	May 2010 and October 2021. Single-institution patients with biopsy-proven MIEC managed with up-front, definitive radiation therapy	55	Median age: 66 years (range, 42-86 years). 12 patients (22%) were diagnosed with HREC. 6 patients (11%) were treated with high-dose-rate brachytherapy alone. 49 patients (89%) were treated with high-dose-rate brachytherapy and external beam radiation therapy. 12 patients (22%) were treated with radiation and chemotherapy. Patient stratification: low-risk endometrial carcinoma (LREC; uterine-confined grade 1-2 endometrioid adenocarcinoma); high-risk endometrial carcinoma (HREC; stage III/IV and/or grade 3 endometrioid carcinoma, or any stage serous or clear cell carcinoma or carcinosarcoma).		44 months (range, 4-135 months)	HDR median CTV D90 EQD2: 53.26 (24.6-92.88). HDR median bladder D2cc EQD2: 51 (36.1-66.22). HDR median rectum D2cc EQD2: 24.9 (6.9-61). HDR median sigmoid D2cc EQD2: 49 (11.3-66.12). HDR median vaginal wall D2cc EQD2: 44.3 (442.7-69.73). HDR + EBRT median CTV D90 EQD2: 77.2 (50.1-175.28). HDR + EBRT median bladder D2cc EQD2: 80.1 (59.5-153.54). HDR + EBRT median rectum D2cc EQD2: 67.75 (48.3-140.75). HDR + EBRT median sigmoid D2cc EQD2: 71.05 (51.4-138.59). HDR + EBRT median vaginal wall. D2cc EQD2: 104.7 (54-173.46)	LREC 2-year OS was 92%. HREC 2-year OS was 80% (log p = 0064)	NA	LREC 2-year CFS was 82%. HREC 2-year CFS was 80% (log rank p = .0654). 2-year CSS was 100% for both LREC and HREC	No acute grade ≥3 toxic effects. 3 late grade ≥3 toxic effects owing to endometrial bleeding and GI adverse effects.
Yaney et al. 2021 [22]	January 2003 and January 2017. Single-institution definitive radiation for MIEC	51	Treated with definitive RT for EC. Majority had endometrioid histology (N = 46, 90.2%) and Grade 1 disease (N = 32, 62.75%). 37 patients (72.5%) were treated with image-guided BT (IGBT) and 14 (27.5%) with two-dimensional BT. 40 patients (78.4%) received EBRT + BT. 11 (21.57%) received BT alone			If EBRT was given, typically two to three fractions of intracavitary brachytherapy on nonconsecutive days were done. If EBRT was not included in the treatment regimen, brachytherapy was typically delivered in four fractions delivered once or twice a week. The goal was to deliver a total EQD2 dose of 80-90 Gy to the GTV, if applicable, and to achieve a CTV D90 of 60-70 Gy. Goals for OARs were to achieve a total EQD2 dose to 2 cc (D2cc) of the rectum and sigmoid less than 75 Gy and the bladder less than 90 Gy.	1- and 2-year OS were 88% and 72% with BT alone. 1- and 2-year OS were 94% and 84%, respectively, with EBRT + BT	1- and 2-year local control were 100% with BT alone. 1- and 2-year local control were 93% and 89%, respectively, with EBRT + BT. 1- and 2-year locoregional control were 100% with BT. 1- and 2-year locoregional control were 97% and 93%, respectively, with EBRT + ВТ.	1- and 2-year RFS was 89% with BT alone. 1- and 2-year RFS were 87% and 80% with EBRT + BT. No statistically significant differences in cancer control between the two groups.	No grade 2 or higher toxicities were reported with BT alone. G2 or higher acute toxicities with EBRT + BT were G2 proctitis (N = 2, 5.0%) and G3 proctitis (N = 1, 2.5%). Late toxicities included G3 vaginal stenosis (N = 1, 2.5%), proctitis (N = 1, 2.5%), enteritis (N = 1, 2.5%), and 1 G4 gastrointestinal bleed.
Mutyala et al. 2021 [20]	Case series retrospective review: 2008 to 2020; MIEC cases treated with definitive image-guided brachytherapy. Single-institution systematic review: Medline (PubMed) from 1975 to 2020	Case series: 31 patients. SR: 19 articles	Case series: 31 cases were included in this study, stages I-IV, with 96.7% receiving external beam radiation. Systematic review: 19 articles were included in the final analysis, with between six and 280 patients.	I, II, III, IV		All patients received three fractions of 7.5 Gy or five fractions of 6 Gy HDR BT, with a median EQD2 of 75.55 (40-84.3).	2-year OS was 77.4%	2-year local control was 83.1%. local control ranged from 70% to 100%, with low toxicity.		No late toxicity ≥ grade 3.
Arians et al. 2020 [16]	2005 and 2018. MIEC patients receiving primary brachytherapy ± external beam radiotherapy (EBRT) via the clinical cancer registry of the National Center for Tumor Diseases (NCT) single institution	13	7 patients received BT alone. 6 patients were treated w/ BT+EBRT. Treated with radiotherapy for endometrial cancer because of medical inoperability (n = 12) or refusal of resection (n = 1). Median age 73.9 years (60.4-87.1 years). 11 patients were staged FIGO IA/B, 1 patient each with FIGO IIIA and IIIC.	IA/B, IIIA/C	78.8 months	EBRT ranging from 45 to 56.5 Gy BT: not reported	2-/5-year-OS were 76.9%/69.2%, respectively.	Estimated 2-/5-year LFFS were 76.2%/56.4%, respectively. High grading correlated with a worse LFFS (p = 0.069).	Estimated 2-/5-year PFS were 76.9%/53.8%	No acute toxicities > grade II and only two late toxicities grade II/III occurred. Three peri-interventional complications.
Espenel et al. 2020 [30]	2002 and 2017. MIEC patients treated with radiotherapy and 3D-based brachytherapy	27	Causes of inoperability were comorbidities (37%) or tumor locoregional extent (63%).		36.5 months (SD = 30.2)	EBRT delivered a dose of 45 Gy. Then, patients had an uterovaginal brachytherapy guided by 3D imaging. Median D90 CTVBT dose was 17.3 Gy EQD2 (IQR = 14.7–22.6 Gy EQD2). Median D90 GTVres dose was 30.0 Gy EQD2 (20.4–40.0 Gy EQD2). EBRT+BT: median D90 CTVBT dose was 60.7 Gy EQD2 (IQR = 56.4-64.2 Gy EQD2), median D90 GTVres dose was 73.6 Gy EQD2 (64.1-83.7 Gy EQD2). Median overall treatment time was 50 days (IQR = 46-54).	5-year OS was 63% (95% CI = 43-91)	Cumulative incidence of local failures was 19% (n = 5). Cumulative incidence of pelvic failures was 7% (n = 2). Cumulative incidence of distant failures was 26% (n = 7), respectively.		Late urinary and GI toxicities ≥ grade 2 were reported in 4 (15%) and 2 patients (7%), respectively. No vaginal toxicity ≥ grade 2 was reported.
Gannavarapu et al. 2020 [18]	April 2012 and January 2019; update in May 2019 MIEC patients who underwent upfront radiotherapy	29	Treated with upfront radiotherapy at an academic medical center from 2012 to 2019. 20 cancers were stage I + Il and 9 were stage Ill. 21 cancers were grade 1 + 2 and 8 were grade 3. 13 patients (45%) had high-risk endometrial cancer (HREC; stage III and/or grade 3). 25 patients received radiotherapy/chemoradiotherapy for primary treatment, 4 patients received chemoradiotherapy before surgery. All patients underwent high-dose rate brachytherapy (HDR): 7 received HDR alone; 22 received external beam radiation and HDR.	I, II, III	17.0 months (range 3.7-54.0)	D90 for HDR-only group: was to deliver an equivalent dose in 2 Gy fractions (EQD2 ) of 48-62.5 Gy to 90% (CTV D90) OARs: These constraints included D2cc bladder ≤80-100 Gy EQD2, D2cc rectum ≤70-75 Gy EQD2, D2cc sigmoid ≤70-75 Gy EQD2, and D2cc small bowel ≤60-65 Gy EQD2. D90 for both EBRT and HDR group was to deliver a D90 EQD2 of 65-75 Gy to the CTV	No OS difference between HREC and LREC patients (2-year: 73% vs. 77%; log-rank p = 0.33).	4 HREC and 1 LREC patient recurred, with 1 local recurrence in each group.	2-year CSS was 100% for both HREC and LREC patients (log-rank p = 0.32).	No acute grade ≥3 GI/GU toxicities. 2 late grade ≥3 GI/GU toxicities
Draghini et al. 2017 [24]	March 2005 and April 2016. MIEC patients treated with definitive HDR-BT with or without EBRT	17	14 (3%) HDR-BT alone, and 3 (1%) EBRT plus HDR-BT without surgery. Median age 79 years (range, 60-95). Median Karnofsky performance status 90% (range, 60-100). Histology was endometrial adenocarcinoma in 14 (82%), and non-endometrial in 3 (18%) patients. In 15 (88%) patients, clinical stage was I, and in the remaining, 2 (12%) was III. All patients were evaluated with computed tomography (CT) and endometrial biopsy.	I, III	53 months (range 6-131)	Median BT CTV 80.6 (range 50-270). 3 patients received EBRT 46-50 Gy + HDR-BT 3-7 x 5-8 Gy. 14 patients received HDR-BT 3-7 x 5-8 Gy	NA	All patients had a clinical local control, after a median follow-up of 53 months (range, 6-131). 3- and 6-year LC rates were 86% and 69%, respectively. Patients with low-risk histology had a better LC at 1 and 6 years compared to those with high-risk histology (100% and 100%, vs 73% and 36%, respectively; p = 0.05). Median duration of LC for low-risk histology was not reached. Median duration of LC for high-risk histology was 73 months	Cancer-specific survival (CSS) at 1, 2, and 6 years were 93%, 85%, and 85%, respectively. DFS at 1, 2, and 6 years were 86%, 79%, and 63%, respectively. Age, stage, dose, and type of radiotherapy did not result significant prognostic factors for LC and CSS. Only histology significantly influenced LC: for high-risk histology (i.e., non-endometrial carcinoma or grade [G] 3 endometrial adenocarcinoma)	Only patients who received both BT + EBRT developed toxicities. Two (12%) patients had G2 acute toxicity and two others (12%) G1 late toxicity.
Dutta et al. 2017 [28]	Systematic review 1992 to 2016; outcomes of MIEC patients treated with radiation alone. National Cancer Database (NCDB) query: 2004 to 2012; to identify patients 65 years and older with Stage I-II MIEC	13 papers, 888 total patients 65 years and older with Stage I-II medically inoperable endometrial cancer		I, II		NA	OS for Stage I tumors at 5 years was 30-95%. EBRT + BT was associated with the most favorable survival; HR of 0.442 (p < 0.001 over no radiotherapy)	Reported pelvic control for the 888 total patients with Stage I tumors was 80-100% and 61-89% for Stage Il	Benefits were also seen with EBRT alone (HR 0.694, p < 0.001) benefits were also seen with BT alone (HR 0.499, p < 0.001) compared to no radiotherapy.	Late complications for all patients treated ranged from 0% to 21% across patients
Jordan et al. 2017 [32]	2010 to 2016. Patients with early-stage EC who underwent primary radiation treatment	15	8 patients received external beam radiation therapy followed by intracavitary HDR brachytherapy. 7 patients underwent intracavitary HDR brachytherapy alone. 9 patients were FIGO. 16 patients were FIGO 2	I, II	29 months	In all patients, mean cumulative D90 to GTV was 95.99 Gy, EQD2 α/β = 10. Mean cumulative D90 EQD2 to CTV was 51.64 Gy.	NA	NA	4 patients died from concurrent diseases at an average of 2.83 years after completion of treatment. Except for 1 (6.6%) patient who recurred at 9 months following completion of treatment, all patients remained disease-free for the remainder of follow-up.	
Acharya et al. 2016 [15]	April 2003 to January 2015. Patients with newly diagnosed MIEC treated with HDR brachytherapy with or without external beam radiation therapy	43	Median BMI was 50.1 kg/m^2^ (range: 25.1-104 kg/m^2^). 36 patients (83.7%) had FIGO clinical Stage I disease, 6 patients (14%) FIGO clinical Stage II disease, and 1 patient (2.3%) FIGO clinical Stage III disease. 28 patients received HDR-BT. 15 patients received HDR-BT + EBRT	I, II, III	29.7 months	HDR brachytherapy alone: median CTV D90 was 30.3 Gy EQD2 (range: 17-40.9). HDR brachytherapy alone: median rectum D2cc and bladder D2cc was 39.8 Gy EQD2 (range: 23.6-53.1) and 44.3 Gy EQD2, respectively. BT+EBRT: median CTV D90 was 37 Gy EQD2 (range: 31-56). BT+EBRT: median rectum D2cc and bladder D2cc was 60.4 Gy EQD2 (range: 52-64.8) and 78.4 Gy EQD2 (range: 49.8-73.5), respectively	2-year overall survival was 65.2% (95% CI: 46.7-78.6). No significant survival difference between BT alone vs BT + EBRT despite high-risk features in the combination group: two-year OS: 69.4% (95% CI: 46.1-84.2%) vs. 57.9% (95% CI: 26-80.1), (p = 0.79)	2-year cumulative incidence of pelvic failure was 8.3%. 2-year cumulative incidence of distant failure was 13.5%. Grade 3 disease was associated with a higher risk of all failures (HR: 4.67, 95% CI: 1.04-20.9, p = 0.044)	There were significant differences in tumor characteristics between patients who were treated with HDR brachytherapy alone compared to those treated with combination therapy. No significant differences based on age, follow-up time, age-adjusted Charlson comorbidity index (CCI) or pretreatment hemoglobin.	Incidence of acute Grade 3 GI/GU toxicities were 4.6%.
van der Steen-Banasik et al. 2016 [4]	January 1969 to 2013. Systematic review of articles in English or French that were published in PubMed. Identified publications on radiation therapy (RT) as single treatment for localized MIEC	25 reports containing 2694 total patients	EBRT combined with BT was used in 1278 patients (47.4%), BT alone in 1383 patients (51.3%), EBRT alone in 33 patients (1.2%)	I, II, III, IV	NA	NA	Combined 5-year OS was 53.2% (95% CI: 49.3%-57.1%)	Combined 5-year LC was 79.9% (range: 75.4-100%; 95% CI: 75.7%-84.1%)	Combined 5-year DSS was 78.5% (range: 68.4-92%; 95% CI: 74.5-82.5)	Average occurrence of grade 3 or worse late toxicity was 3.7% for EBRT + BT, compared to 2.8% for BT alone, and 1.2% for EBRT alone. Average incidence of severe complication requiring surgery was 1.6% (range: 0-5%)
Gill et al. 2014 [31]	2007 to 2013. MIEC clinical Stage I, who received definitive BT with or without external beam radiotherapy	38	20 received BT alone. 18 received BT + EBRT		15 months	BT alone: median dose of 37.5 Gy in five to six fractions. EBRT + BT: median external beam and BT doses were 45 and 25 Gy, respectively, in four to five fractions. Mean CTV D90 EQD2 for BT alone: 48.6 ± 5.6. Mean GTV D90 EQD2 for BT alone: 172.3 ± 59.6. Mean CTV D90 EQD2 for combined therapy: 72.4 ± 6.0 Gy. Mean GTV D90 EQD2 for combined therapy: 138.0 ± 64.6 Gy.	2-year overall survival was 94.4%	2-year local control was 90.6%	NA	No Grade 2-5 late toxicities were observed.

The strength of our study is that it reinforces the efficacy and safety of definitive brachytherapy with EBRT for the treatment of MIEC, a group of patients that can be expected to increase over the next decade. The limitations of this study include its small sample size and retrospective evaluation. Another limitation is that multiple patients in our sample were referred to our center for brachytherapy from outside facilities, with radiation treatment delivered by multiple physicians, thus reducing the consistency in radiation treatment delivery.

## Conclusions

Endometrial cancer has been rising in both incidence and mortality in the United States for over 30 years, as significant risk factors such as increased lifespan, obesity, and type 2 diabetes have also been rising. Thus, it is reasonable to assume that the incidence of MIEC will also increase, making a need to optimize treatment for this group of patients. Brachytherapy with EBRT can be used to treat this subset of patients with reasonable survival and minimal toxicity. Future research is needed to evaluate definitive radiation therapy combined with novel systemic therapy, such as immunotherapy, and to assess outcomes with different brachytherapy applicators for MIEC.
